# Identification of biomarkers and pathogenesis in severe asthma by coexpression network analysis

**DOI:** 10.1186/s12920-021-00892-4

**Published:** 2021-02-18

**Authors:** Zeyi Zhang, Jingjing Wang, Ou Chen

**Affiliations:** School of Nursing and Rehabilitation, Cheeloo College of Medicine, Shandong University, #44 West Wenhua Road, Jinan, 250012 China

**Keywords:** Severe asthma, Gene expression, Pathogenesis, WGCNA

## Abstract

**Background:**

Severe asthma is a heterogeneous inflammatory disease. The increase in precise immunotherapy for severe asthmatics requires a greater understanding of molecular mechanisms and biomarkers. In this study, we aimed to identify the underlying mechanisms and hub genes that determine asthma severity.

**Methods:**

Differentially expressed genes (DEGs) were identified based on bronchial epithelial brushings from mild and severe asthmatics. Then, weighted gene coexpression network analysis (WGCNA) was used to identify gene networks and the module most significantly associated with asthma severity. Furthermore, hub gene screening and functional enrichment analysis were performed. Replication with another dataset was conducted to validate the hub genes.

**Results:**

DEGs from 14 mild and 11 severe asthmatics were subjected to WGCNA. Six modules associated with asthma severity were identified. Three modules were positively correlated (*P* < 0.001) with asthma severity and contained genes that were upregulated in severe asthmatics. Functional enrichment analysis showed that genes in the most significant module were mainly enriched in neutrophil activation and degranulation, and cytokine receptor interaction. Hub genes included CXCR1, CXCR2, CCR1, CCR7, TLR2, FPR1, FCGR3B, FCGR2A, ITGAM, and PLEK; CXCR1, CXCR2, and TLR2 were significantly related to asthma severity in the validation dataset. The combination of ten hub genes exhibited a moderate ability to distinguish between severe and mild-moderate asthmatics.

**Conclusion:**

Our results identified biomarkers and characterized potential pathogenesis of severe asthma, providing insight into treatment targets and prognostic markers.

**Supplementary Information:**

The online version contains supplementary material available at 10.1186/s12920-021-00892-4.

## Introduction

Asthma is a chronic, heterogeneous inflammatory disease with complex pathological mechanisms and diverse clinical phenotypes. Severe asthma is one of the phenotypes, which is defined as uncontrolled asthma despite adherence to maximally optimized therapy and asthma worsens when high-dose treatment is decreased [1]. Patients with severe asthma attempt to achieve control and prevent life-threatening exacerbations with high doses of inhaled corticosteroids or even oral corticosteroids [2], with a 3.1-fold higher risk of developing osteoporotic fracture and a 2.7-fold higher risk of developing pneumonia [3]. Furthermore, corticosteroid resistance is common in severe asthma patients, making corticosteroid therapy less effective [4, 5]. Considering the side effects and limitations of traditional therapies, novel treatments focusing on the immune system have been were developed. Nevertheless, early attempts at immunosuppressive therapies have been unsuccessful [2], underlining a need for a comprehensive understanding of molecular mechanism and endotypes of severe asthma.

Weighted gene coexpression network analysis (WGCNA) is a bioinformatics method for exploring the complex relationships between gene expression profiles and phenotypes. WGCNA is widely used in studies of multigene diseases to identify potential biomarkers and provide molecular targets for treatment. Some researchers also used this method to explore the pathogenesis of asthma and identify pathways and genes associated with asthma severity [6–8]. Nevertheless, differentially expressed genes (DEGs) between healthy controls and asthma patients or genes from all asthmatics, not DEGs between mild and severe asthmatics, were considered to construct a coexpression network in the studies mentioned above. Analysis of DEGs from mild-severe asthmatics could identify genes that especially contribute to disease progression. In this study, such genes were considered for WGCNA and further biologically functional analysis to define hidden mechanisms and key genes in severe asthmatics. The results will shed light on treatment targets and inform the prognosis assessment of severe asthma.

## Materials and methods

### Data processing and differential gene expression analysis

Dataset related to severe asthma was obtained from the Gene Expression Omnibus (GEO) datasets (https://www.ncbi.nlm.nih.gov/gds) with accession number GSE89809 [9]. Platform information was GPL13158. This dataset contains 145 samples of different tissue types (i.e., bronchoalveolar lavage, sputum, epithelial brushings) from healthy controls, mild, moderate, and severe asthmatics. Asthma was defined according to GINA 2012 [9]. Asthma severity was assessed using previously described criteria [10]. As bronchial epithelial cells are thought to be highly informative for describing changes in gene expression in asthma [11, 12], data of epithelial brushings from 14 mild and 11 severe asthmatics were extracted for WGCNA. Accessible clinical traits, including asthma severity, asthma control questionnaire (acq) score, smoking, allergic rhinitis, nasal polyps, inhaled corticosteroid (ICS) dose, FEV1, FVC, reversibility, and GINA control, were analyzed in WGCNA. Raw microarray gene expression data were normalized using RMA method via the R Bioconductor package affy [13] and subjected to several quality control procedures. Then gene IDs were mapped to the microarray probes using annotation information. Probes matching more than one gene were eliminated from the dataset, and the mean expression value of genes measured by multiple probes was calculated. DEGs between severe and mild asthmatics were identified using the limma package in R software [14]. A gene with log two-fold Change > 0.5 and *P* value adjusted by false discovery rate < 0.05 was considered significantly differentially expressed.

### Construction of coexpression modules

The WGCNA package [15] was used to construct a scale-free coexpression network using the obtained DEGs to examine their associations with clinical variables. The soft-thresholding power β was calculated in the construction of each module using the pickSoftThreshold function of WGCNA, which provides a suitable power value for network construction by calculating the scale-free topology fit index for a set of candidate powers that ranges from 1 to 20. If the index value for the reference dataset exceeded 0.85, the appropriate power was determined. The hclust function was used to cluster samples and check for outliers. Then, a one‐step network construction method was used to identify coexpression modules, and the minimum number of genes for each module was set to 50.

The relationships between modules and asthma severity, as well as other clinical traits, were assessed. As the relationship between gene expression and asthma severity may potentially be influenced by some sample-specific traits (e.g. corticosteroid and smoking), a linear model adjusted for confounders was used to confirm the findings.

### Identification of the clinically significant module and hub genes

Module eigengene (ME) represents the first principal component of a given module and the gene expression profiles in this module. Gene significance (GS) and module membership (MM) were defined as the absolute value of the correlation between a gene and a clinic trait and the correlation of gene expression with the ME, respectively. The clinically significant module for asthma severity was identified if:

1. the correlation between the module and asthma severity ≥ |0.5|;
2. the correlation between MM and GS in the module was statistically significant (*P* < 0.05).

The key module was visualized using STRING (version 11.0).

Hub genes are those genes in clinically significant modules that tend to have high connectivity. Genes with |MM| > 0.6 and |GS| > 0.5 in the key module were imported to Cytoscape (version 3.8.2), and then, the top 10 degree genes were filtered as the hub genes. To test whether the hub genes were specific for asthma, the correlation of hub genes and asthma susceptibility was calculated.

### Enrichment analysis

To further classify and visualize the functions of genes in the key module, Gene Ontology (GO) and Kyoto Encyclopedia of Genes and Genomes (KEGG) [16, 17] enrichment analyses were performed on genes in the key module, using the R package clusterProfiler [18]. A *P* value < 0.05 was considered the cutoff criterion. The top 8 categories identified with GO and KEGG analyses were shown.

### Validation of hub genes

Hub genes were validated using the GSE43696 dataset with platform GPL6480 [19, 20]. This dataset provides data from bronchial epithelial brushings from 20 healthy controls, 50 mild-moderate asthmatics and 38 severe asthmatics. Mild and moderate subjects comprised a group that did not use exactly the same criteria as in GSE89809. Severe subjects met American Thoracic Society definitions similar to those in GSE89809 (Additional file 1: Table S3). Data from mild-moderate and severe subjects were used for validation. Subsequently, the dataset was normalized and processed as performed for GSE89809. Expression data of ten hub genes and severity of asthmatics were extracted. The correlation of individual hub genes and asthma severity, as well as the expression of hub genes between mild-moderate and severe asthmatics were analyzed. In addition, receiver operating characteristic (ROC) curve analysis was conducted for each hub gene and combined hub genes with the ROC package [21]. The area under the curve (AUC) was used to evaluate the sensitivity and specificity of the ten hub genes.

## Results

### Dataset selection and DEGs identification

The microarray gene expression dataset GSE89809 was used in this study. After data normalization and quality control, 1035 genes were identified as DEGs, of which 634 were upregulated and 401 were downregulated between mild asthma and severe asthma. A volcano plot of all probesets and a heatmap of the top 25 changed genes were shown in Additional 1: Figure S1-S2.

### Co-expression network construction and disease-specific module identification

**Fig. 1:**
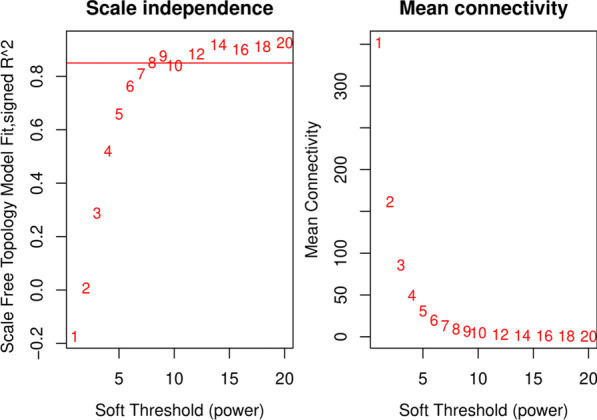
Analysis of network topology for a set of soft‐thresholding powers

**Fig. 2:**
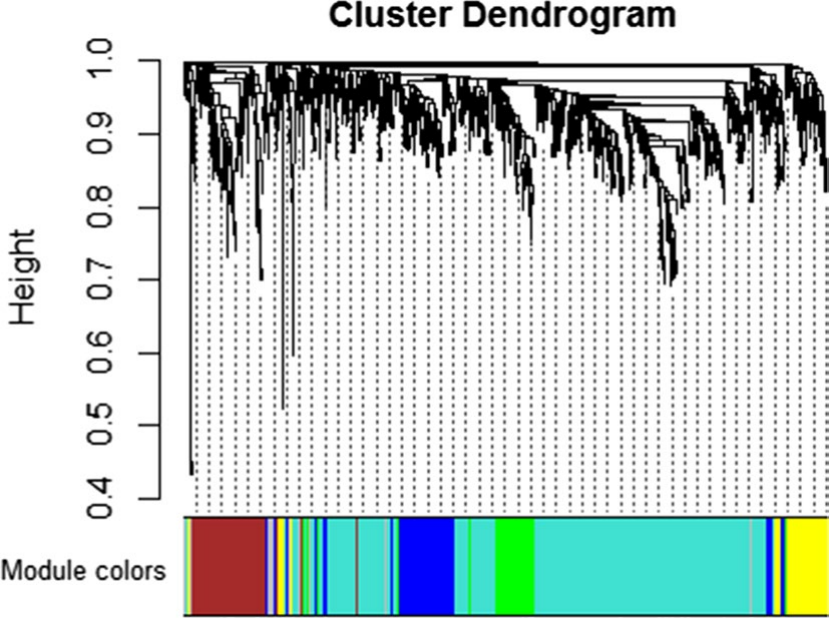
Clustering dendrograms of genes

The expression profiles of 1035 DEGs were used to conduct WGCNA. Hierarchical clustering analysis was then performed. When the threshold was set to 50, GSM2389953 was considered to be an outlier and removed prior to further analyses (Additional 1: Figure S3). The optimal power β for which the scale‐free topology index exceeded 0.85 was computed as 8 (Fig. 1). After this soft threshold of 8 was implemented, 6 significant gene modules, ranging in size from 27 to 585 genes, were detected (Fig. 2). The gray module contained DEGs that did not cluster in any module.

### Identification of the clinically significant module and hub genes

**Fig. 3:**
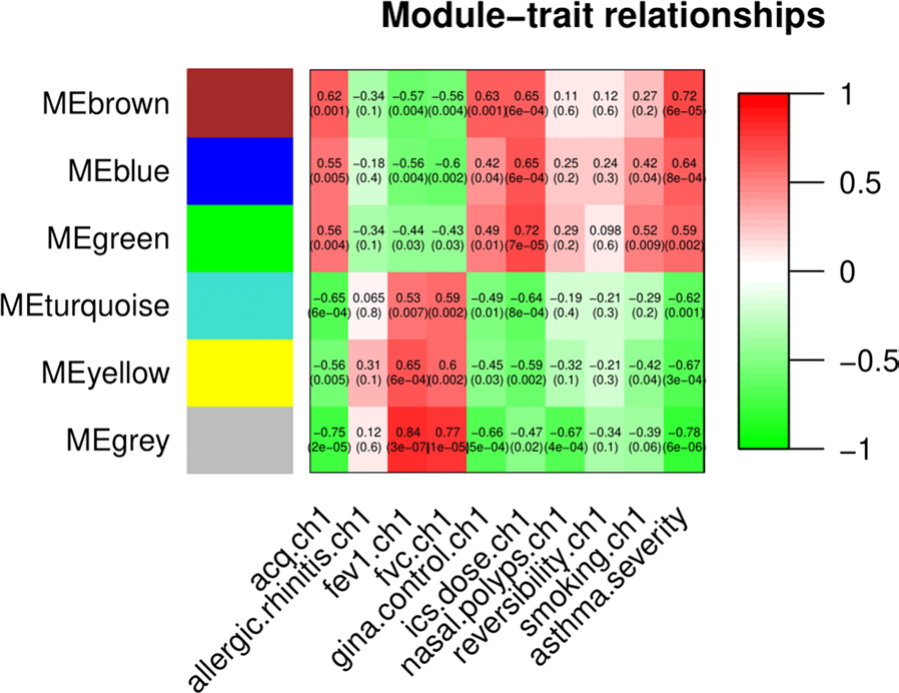
Module‐trait relationships

Module-trait associations were identified based on the correlation between module eigengene and clinical traits. The results (Fig. 3) indicated that all modules were significantly associated with asthma severity. Three modules (brown, blue, and green) were positively correlated with asthma severity, ACQ score, and GINA control, while negatively correlated with FEV1 and FVC. This means that genes in those modules are predominantly upregulated in severe asthma. In contrast, two modules (yellow and turquoise) were found to be negatively correlated with asthma severity, ACQ score, and GINA control. In addition, modules positively associated with asthma severity were also correlated with inhaled corticosteroid (ICS) dose and smoking status. No modules were found to be correlated with allergic rhinitis or nasal polyps. Additional 1: Figure S4 showed the association between modules and asthma severity adjusted by ICS dose and smoking. Both before and after adjustment, the brown module strongly correlated with asthma severity, followed by the yellow module. The eigengene dendrogram and heatmap showed interactions of modules (Additional 1: Figure S5).

**Fig. 4:**
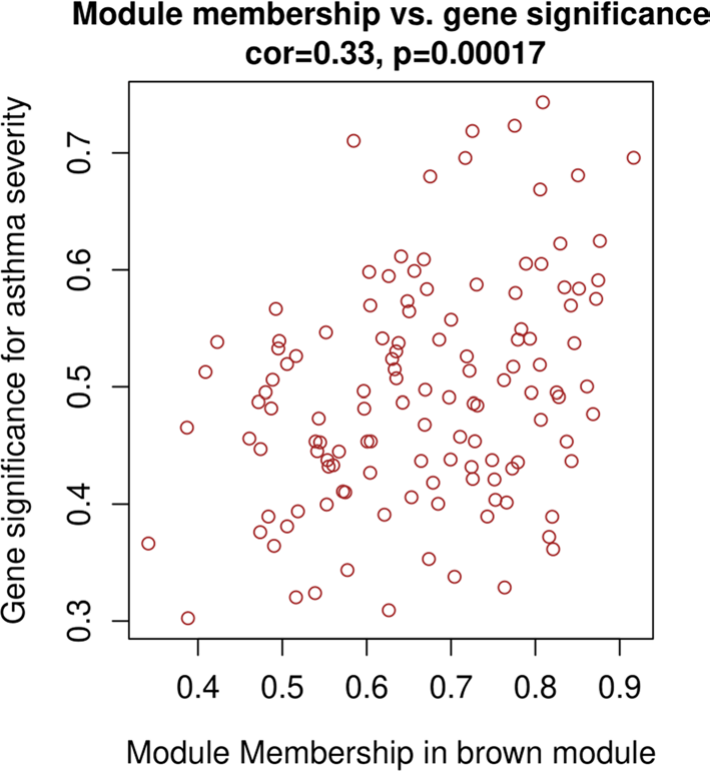
Gene significance for asthma severity vs module membership in brown module

**Fig. 5:**
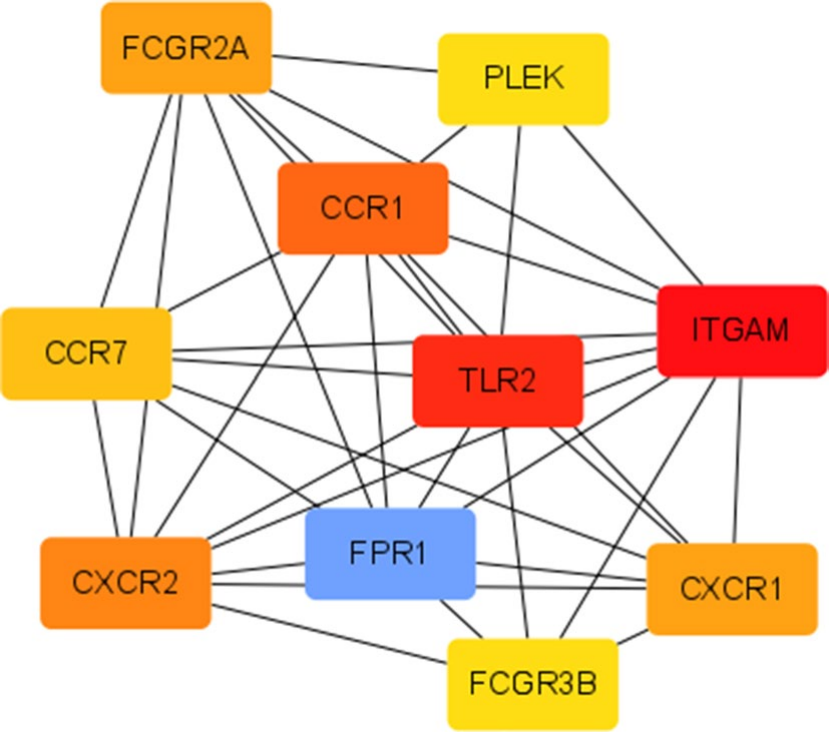
Hub genes visualized in Cytoscape

Among these modules, the brown module had the strongest correlation with asthma severity (r = 0.72, *P* < 0.001), even after adjusting for confounders (adjusted *P* = 0.03). The GS and MM of the brown module were further calculated using WGCNA. Figure 4 showed that the brown module had a strong GS-MM correlation (*P* < 0.001), which was identified as the clinically significant module and visualized in String (Additional 1: Figure S6). In total, 48 genes with |GS| > 0.5 and |MM| > 0.6 in the brown module were imported into Cytoscape, and the top 10 degree genes, namely, CXCR1, CXCR2, CCR1, CCR7, TLR2, FPR1, FCGR3B, FCGR2A, ITGAM and PLEK, were filtered as hub genes (Fig. 5). The correlations of hub genes and asthma susceptibility were further analyzed, considering susceptibility as a dichotomous variable (control vs asthma). The results (Additional 1: Table S1) demonstrated that hub genes were not associated with asthma susceptibility and were specific for asthma severity, which is consistent with our intention of using DEGs from mild-severe asthmatics. Given that other modules were also related to asthma severity before adjustment, those modules and their top genes were shown (Additional 1: Figure S7-S8).

### Functional enrichment analysis

**Fig. 6:**
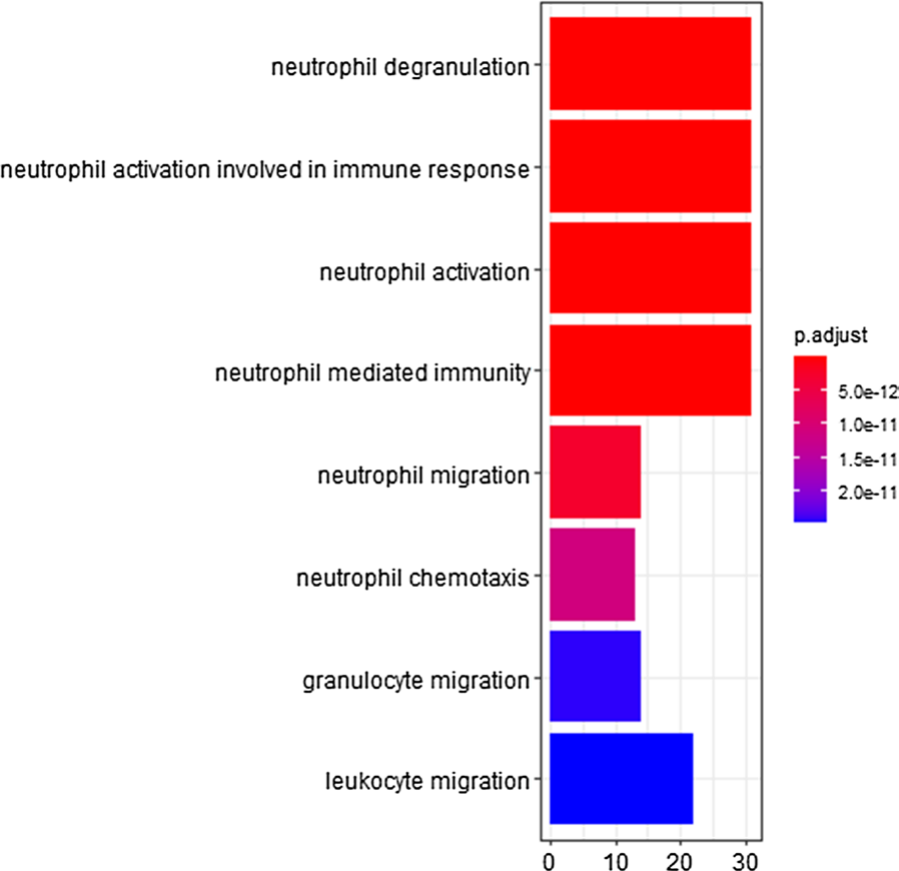
GO enrichment analysis of genes in brown module

**Fig. 7:**
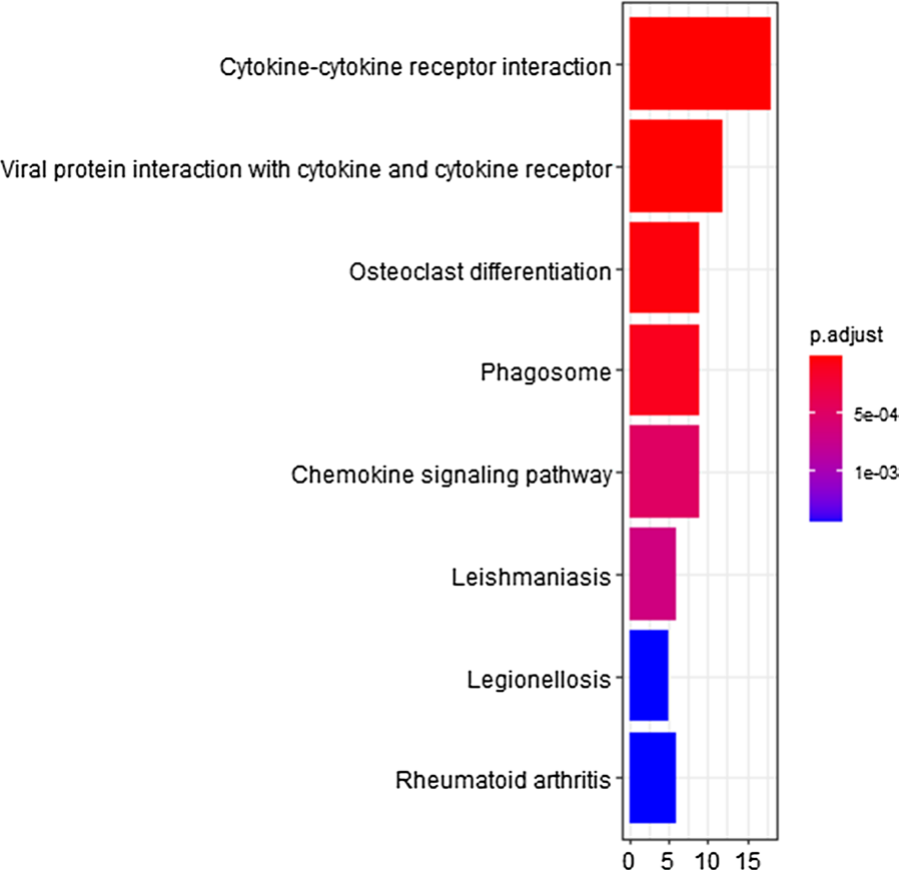
KEGG enrichment analysis of genes in brown module

GO and KEGG enrichment analyses of gene modules were conducted. GO enrichment results showed that the brown module genes were significantly associated with immune responses such as neutrophil degranulation and activation, leukocyte migration and chemotaxis (Fig. 6). The KEGG pathway enrichment results indicated that genes in the brown module were primarily enriched in cytokine–cytokine receptor interaction, phagosome, chemokine signaling pathway (Fig. 7).

In the analysis of interactions between the 5 modules, modules positively related to asthma severity (brown, blue, green module) were correlated with each other. Thus, enrichment analysis was also conducted for genes in positively related modules. The Go and KEGG enrichment results of these modules were in consistent with the results of the brown module (Figure S9).

### Validation of hub genes

**Fig. 8:**
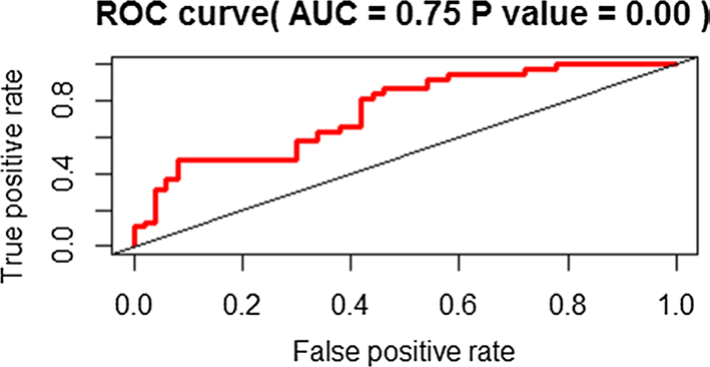
Receiver operating characteristics curve analyses of combined hub genes

To verify hub gene expression, dataset GSE43696 was retrieved from GEO. The correlation analysis results showed that CXCR1 (*P* = 0.02), CXCR2 (*P* = 0.02), and TLR2 (*P* = 0.01) were significantly related to asthma severity in GSE43696 (Additional 1: Table S2). Expression differences of hub genes between groups showed similar results (Additional 1: Figure S10). ROC curve analysis indicated that the AUC for CXCR2 was 0.66 (*P* = 0.01), followed by CXCR1, TLR2, FPR1, FCGR3B, CCR1, and ITGAM (Additional 1: Figure S11). The combination of 10 hub genes possessed a moderate ability to discriminate between severe and mild-moderate asthmatics with an AUC of 0.75(*P* < 0.01) (Fig. 8). ROC curves of top genes in other modules were presented in the supplement (Additional 1: Figure S12).

## Discussion

Severe asthma contributes to 50–60% of asthma costs and is associated with poor quality of life and high mortality and morbidity [22]. The unclear molecular mechanism and refractory response to traditional asthma therapies seen in these patients have been challenging for clinicians to treat this asthma subtype. In this study, for the first time, we used the DEGs between mild and severe asthma samples to construct a coexpression network by WGCNA and carried out a comprehensive analysis of key genes and pathological processes associated with asthma severity, hoping that the findings will be beneficial for the understanding and future treatment of severe asthma.

In total, 6 modules were identified in this paper, of which 3 modules were positively related to asthma severity while 3 modules were negatively related to asthma severity. The brown module, which had the strongest relation to asthma severity and the most significant MM-GS correlation, was identified as the critical module. Enrichment analysis showed that genes in the brown module were enriched in neutrophil degranulation and activation, leukocyte migration and chemotaxis, cytokine-cytokine receptor interaction, phagosome, and chemokine signaling pathways. Then 10 hub genes in the brown module were filtered and verified in another dataset.

The results of module-trait relationships showed that modules positively related to asthma severity (brown, blue and green modules) were also positively related to ACQ score and GINA control grade, but negatively related to FEV1 and FVC, which means that the higher level of gene expression is in these modules, the worse asthma control and lung function are. In addition, positive relationships were found between smoking and upregulated genes in severe asthmatics. Previous studies have shown that asthma patients exposed to smoke are typically steroid-refractory and result in uncontrolled asthma [23]. One of the probable mechanisms has been linked to the Th17 pathway [24], which mediates neutrophilic activation and recruitment in airway. This is consistent with our enrichment analysis results that genes positively to smoking status were enriched in neutrophil degranulation, activation and migration. ICS dose and smoking status may affect the module-asthma severity association. However, adjustments for confounders directly tied to asthma severity (i.e., ICS dose) could mask true biological findings [8] and lead to model overfitting. Therefore, results after adjustment were used to compare with and verify the findings before adjustment. The brown module was significantly related to asthma severity even when confounders were considered, which made it the critical module.

For brown module genes, the significantly enriched terms in GO and KEGG analyses were as follows: neutrophil degranulation and activation, leukocyte migration and chemotaxis, cytokine-cytokine receptor interaction, phagosome, chemokine signaling pathway. Similar enrichment results were found when all modules positively related to asthma severity were considered. Neutrophil inflammation, characterized by the lack of Th2-mediated inflammatory response and increased numbers of neutrophils in the airway [25], has been linked to asthma severity [26, 27], regardless of whether asthma is eosinophilic or noneosinophilic [28, 29]. The original paper using the same dataset also associated neutrophils with asthma severity through a protein interaction network [9]. In our study, visualized GO enrichment analysis further detailed and emphasized the role of neutrophils, with the first six GO terms associated with neutrophils. This means that more neutrophils become activated, degranulate, and migrate as asthma progresses from mild to severe. Furthermore, neutrophil inflammation is more prominent in patients who fail to respond to inhaled corticosteroids, also referred to as severe asthmatics, than in other asthmatics [30]. Thus, novel treatments aimed at decreasing neutrophils may benefit patients with severe asthma. In addition, results of KEGG analysis showed increased cytokine production and functioning in severe asthma, which is reflected by the functions of hub genes.

Identified hub genes further provided biomarkers for severe asthma. The study by Singhania et al. [9] provided evidence of a role of IL-8, which is an essential chemokine that enhances neutrophil migration into airways and contributes to asthma severity and lung damage [31]. In our study, CXCR1 and CXCR2 were further identified as the related chemokine receptors that respond to IL-8. In this way, CXCR1/2 inhibition might be a rational therapeutic strategy for severe asthma treatment. For example, a selective CXCR2 antagonist named SCH527123 was reported to reduce sputum neutrophils and mild exacerbations [32]. However, AZD5069, which is also an antagonist of CXCR2, failed to reduce asthma exacerbations or improve lung function compared with placebo [33]. Recently, a study suggested that KLF2, as a regulator of CXCR1/2, may represent an indicator of asthma severity when combined with CXCR1/2 [34]. This provides another direction for the treatment of severe asthma targeting CXCR1/2.

The toll-like receptor (TLR) family is the first line for defensing against invading microbes [35]. Increased expression of TLR2 was reported in severe asthmatics when compared with healthy controls [9]. Furthermore, when compared with mild asthmatics in our study, severe asthmatics also showed upregulated TLR2, highlighting the role of TLR2 on asthma progression. TLR2 probably take part in asthma progression by inducing Th17 responses and production of IL-8 and IL-17, which could modify airway structures, leading to airway obstruction and low FEV1 seen in patients with severe asthma [36]. In the study by Singhania et al., in fact, IL-17-inducible chemokines were highly expressed across all asthmatics relative to healthy controls, and increased with the increase in asthma severity. Therefore, TLR2 could be linked to asthma severity through the IL-17 pathway. However, a recent study showed that TLR2 may reduce Th17 cytokines by suppressing a Th17 phenotype of Treg cells. This means that TLR2 may induce remission of asthma [37]. Another study in mice also suggested that appropriate stimulation of the TLR2/4 pathway may help to prevent asthma in offspring [38]. Thus, further studies are needed to reveal the effect of TLR2 on asthma and disease severity.

CCR1 and CCR7 are chemokine receptors in Th2/type 2 pathway, which is thought to be the dominant inflammatory pathway underlying severe asthma. CCR1 is mainly expressed in eosinophils, macrophages, and lymphocytes. CCR1 plays a role in the progression of asthma by promoting the chemotaxis of leukocytes in the airway epithelium and probably by modulating the balance of Th1/Th2 cytokine [39]. Biopsies of airways have demonstrated elevated expression of CCR1 mRNA in mild-to-severe asthma [40]. CCR7 is involved in the migration and maturation of dendritic cells (DCs), which could facilitate the development of asthma [41–43]. CCR7 could also participate in the airway remodeling of severe asthma by enhancing fibrocyte transmigration [44]. In addition, findings about the role of CCR7 in immune tolerance in allergy-induced asthmatics [45, 46] may provide ideas for the treatment of severe asthma.

FPR1 is a powerful neutrophil chemotactic factor and has been linked to chronic inflammatory diseases. Although FPR1 was reported to react to cigarette smoke [47, 48] and be involved in the anti-inflammatory activities of glucocorticoids [49], little is known about its effect on asthma. FCGR3B, FCGR2A and ITGAM are immune-related genes, all of which are known as biomarkers for systemic lupus erythematosus. PLEK is a major protein kinase C substrate of platelets, monocytes, macrophages and lymphocytes. The exact function of these genes in asthma patients is not known.

In this study, hub genes identified from GSE89809 were validated in another dataset. However, some of these genes were not related to asthma severity in GSE43696. This may occur due to the dissimilar grouping between the two datasets, as mild and moderate asthmatics were included in a group in GSE43696. Nevertheless, the relationship between CXCR1, CXCR2, TLR2 and asthma severity was stable, and combined hub genes were able to discriminate severe asthmatics from mild-moderate asthmatics in ROC analysis.

For the first time, coexpression modules were built via WGCNA using DEGs of mild-severe asthmatics from bronchial epithelial brushings to discover mechanisms and hub genes in severe asthma. There are some limitations in our study. First, the sample size was small, which may affect the stability of the findings. Although replication was performed to reduce this issue, the results should be interpreted carefully. In addition, the validation dataset GSE43696 used different grouping criteria than those used in GSE89809. Moreover, cell type, ICS dose and smoking may influence gene expression and module-trait relationships. Considering this issue, we adjusted for confounders to verify our findings. Finally, our research is based on public online database information. Future experiments are required to elucidate the detailed mechanisms of the identified hub genes and prove the results.

In conclusion, we identified neutrophil degranulation and activation as key pathways in the asthma progression. Furthermore, hub genes, such as CXCR1, CXCR2, and TLR2, were identified as biomarkers of asthma severity through either the neutrophil inflammation pathway or Th17 immune pathway. Our results can be useful to provide potential immunotherapy targets and prognostic markers. Further mechanistic studies are required to validate and elucidate our results.

## Supplementary Information

**Additional file 1.** Supplementary figures and tables on modules and genes.

## Data Availability

Datasets generated and analyzed during the current study are available in Gene Expression Omnibus (GEO), (https://www.ncbi.nlm.nih.gov/gds/).
