# Supplementary material for: Identification of biomarkers and pathogenesis in severe asthma by coexpression network analysis
Source: BMC Med Genomics. 2021 Feb 18;14:51. doi: 10.1186/s12920-021-00892-4 (PMC7893911; doi:10.1186/s12920-021-00892-4)
Supplement: Supplementary file 1 — Additional file 1. Supplementary figures and tables on modules and genes. [file 12920_2021_892_MOESM1_ESM.docx]

**Identification of Biomarkers and Pathogenesis in Severe Asthma by Coexpression Network Analysis**

Zeyi Zhang, Jingjing Wang, Ou Chen^*^

Author affiliations: School of Nursing and Rehabilitation, Cheeloo College of Medicine, Shandong University

#44 West Wenhua Road, Jinan250012, China

First author: Zeyi Zhang

Email: Zhzeyi@outlook.com

*Correspondence: Ou Chen

School of Nursing and Rehabilitation, Shandong University, #44 West Wenhua Road, Jinan 250012, China

Tel **:** 86+15990991181

Fax **:** 0531-88382201

Email ：[chenou@sdu.edu.cn](mailto:chenou@sdu.edu.cn)

**Content:**

Figure S1. Volcano plot of all probesets

Figure S2. Heatmap of the top 25 changed genes

Figure S3. Hierarchical clustering analysis

Figure S4. Relationships between modules and asthma severity adjusted by confounders

Figure S5. The eigengene dendrogram and heatmap

Figure S6. Brown module visualized in String database

Figure S7. Modules visualized in String database

Figure S8. Top genes of modules visualized in Cytoscape

Figure S9. Results of GO and KEGG enrichment analysis of genes in modules positively related to asthma severity

Figure S10. Expression of hub genes between mild-moderate asthma and severe asthma

Figure S11. Receiver operating characteristics curve analyses of hub genes in GSE43696

Figure S12. Receiver operating characteristics curves of combined top genes in each module

Table S1. Correlations of hub genes and asthma

Table S2. Correlation of hub genes and asthma severity in GSE43696

Table S3. Definitions of the Participants between GSE89809 and GSE43696


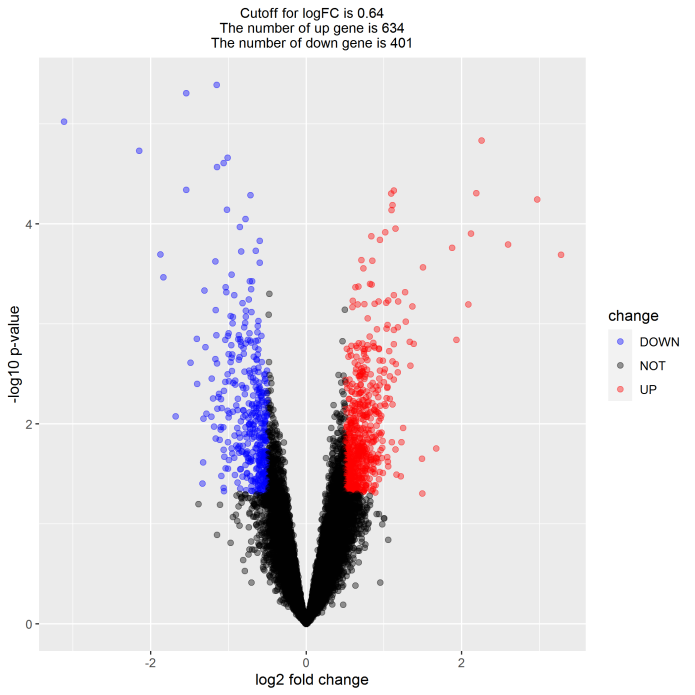


Figure S1. Volcano plot of all probesets


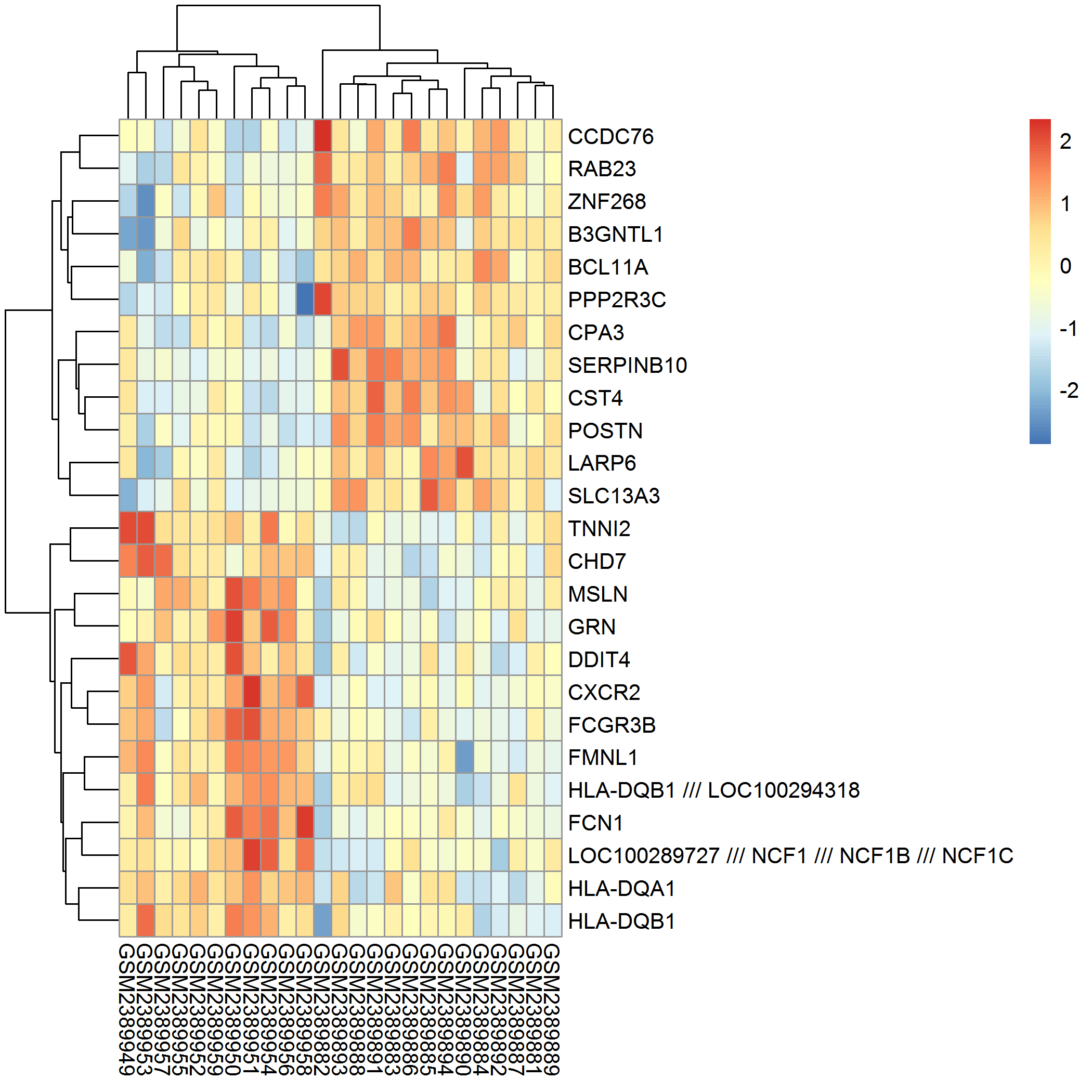


Figure S2. Heatmap of the top 25 changed genes


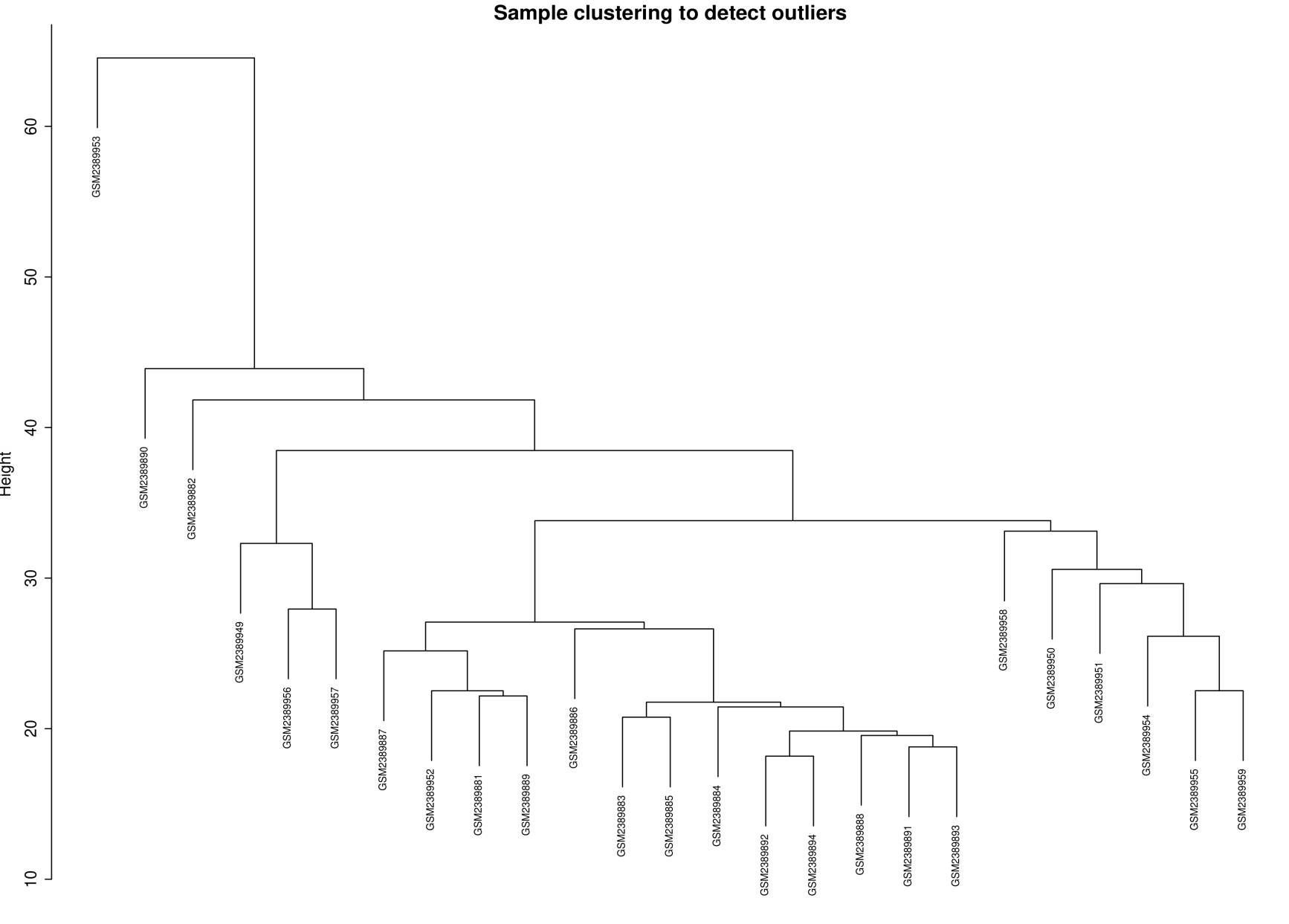


Figure S3. Hierarchical clustering analysis


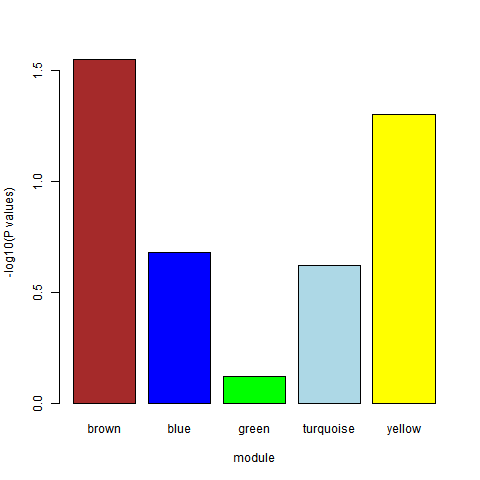


Figure S4. Relationships between modules and asthma severity adjusted by confounders


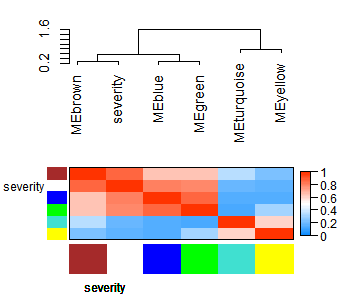


Figure S5. The eigengene dendrogram and heatmap


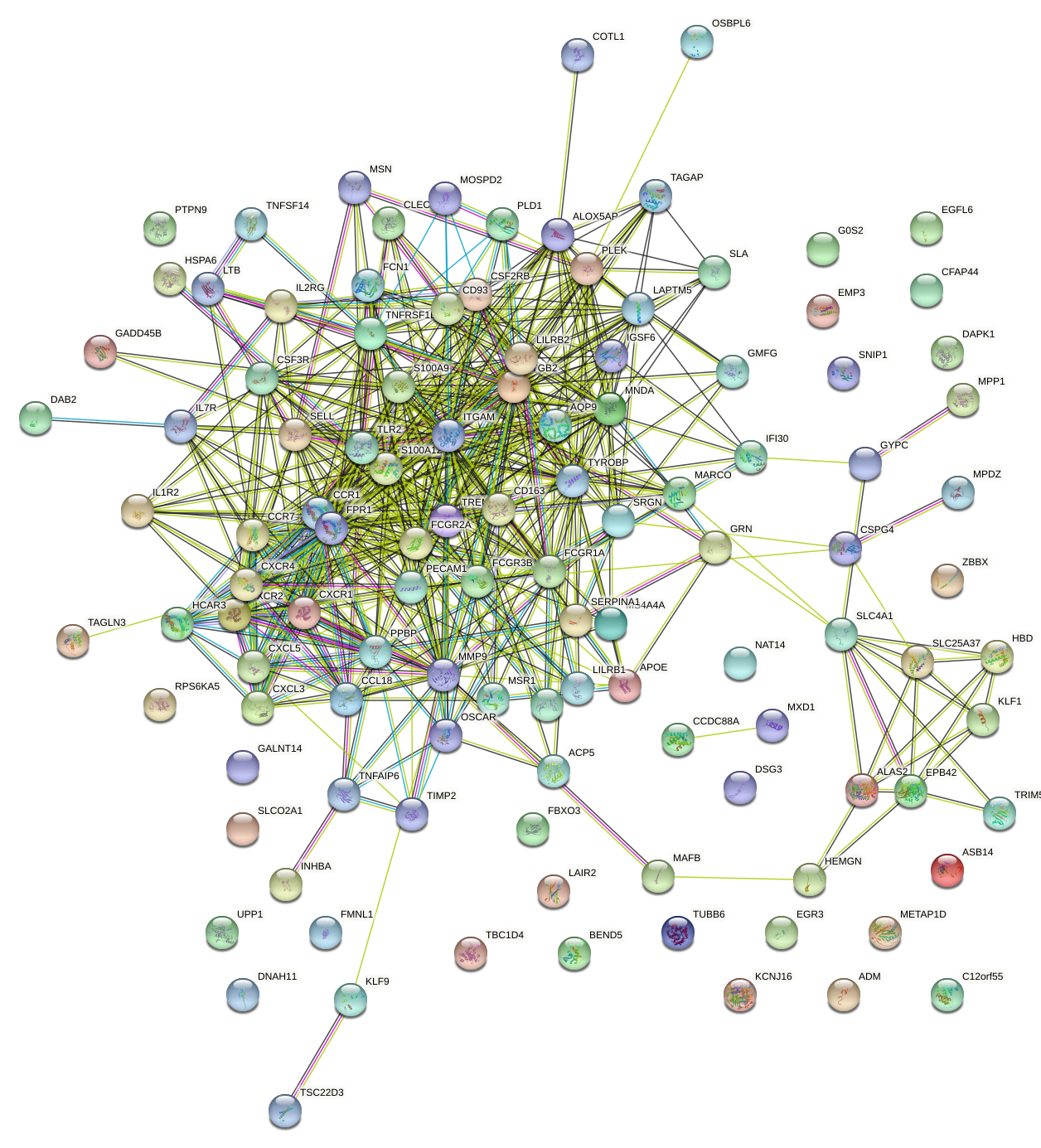


Figure S6. Brown module visualized in String database


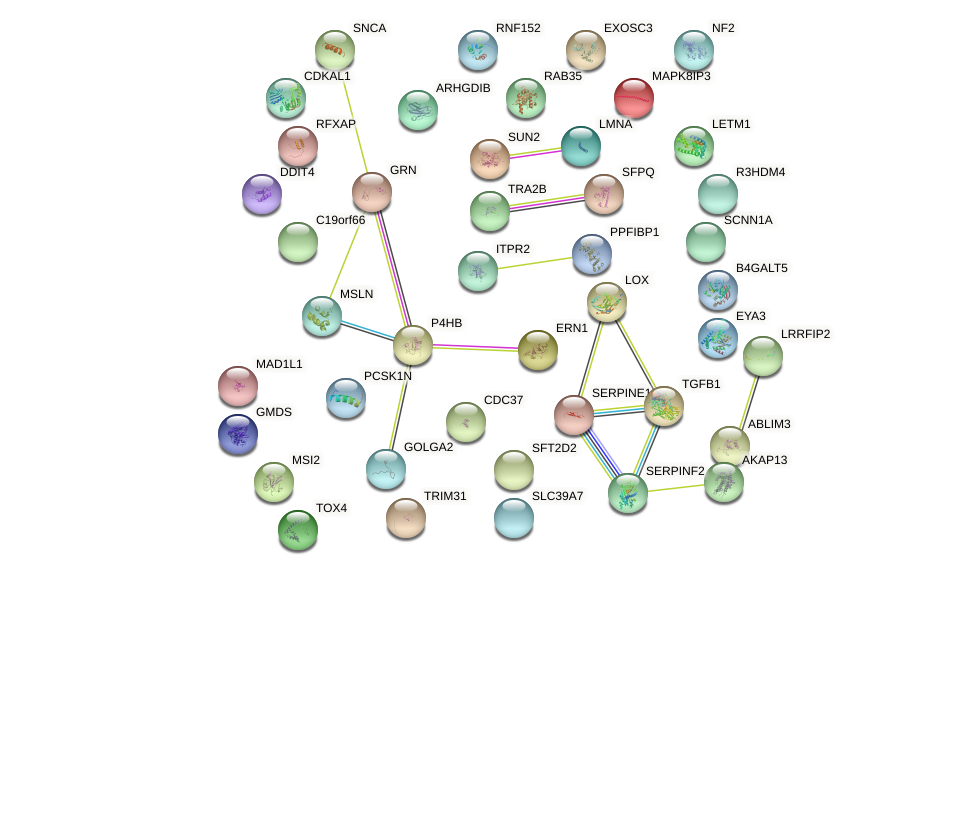

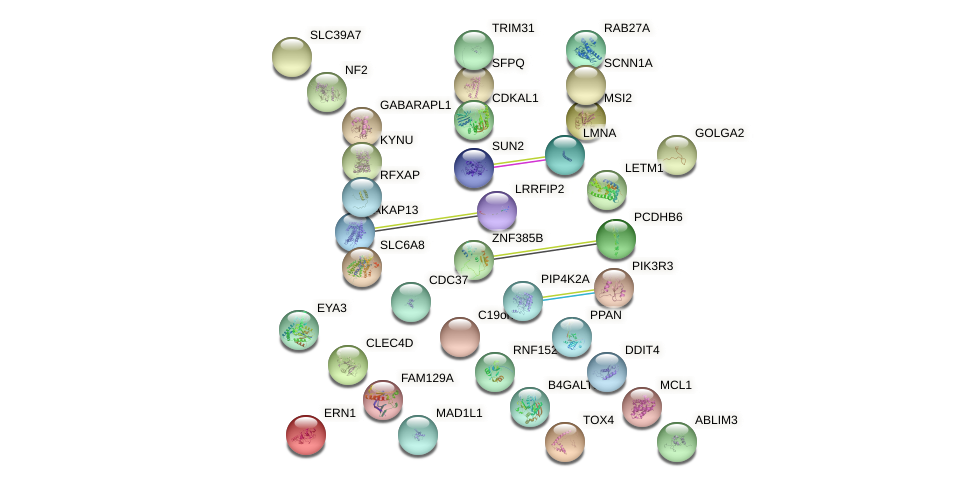


**A**

**B**

**C**


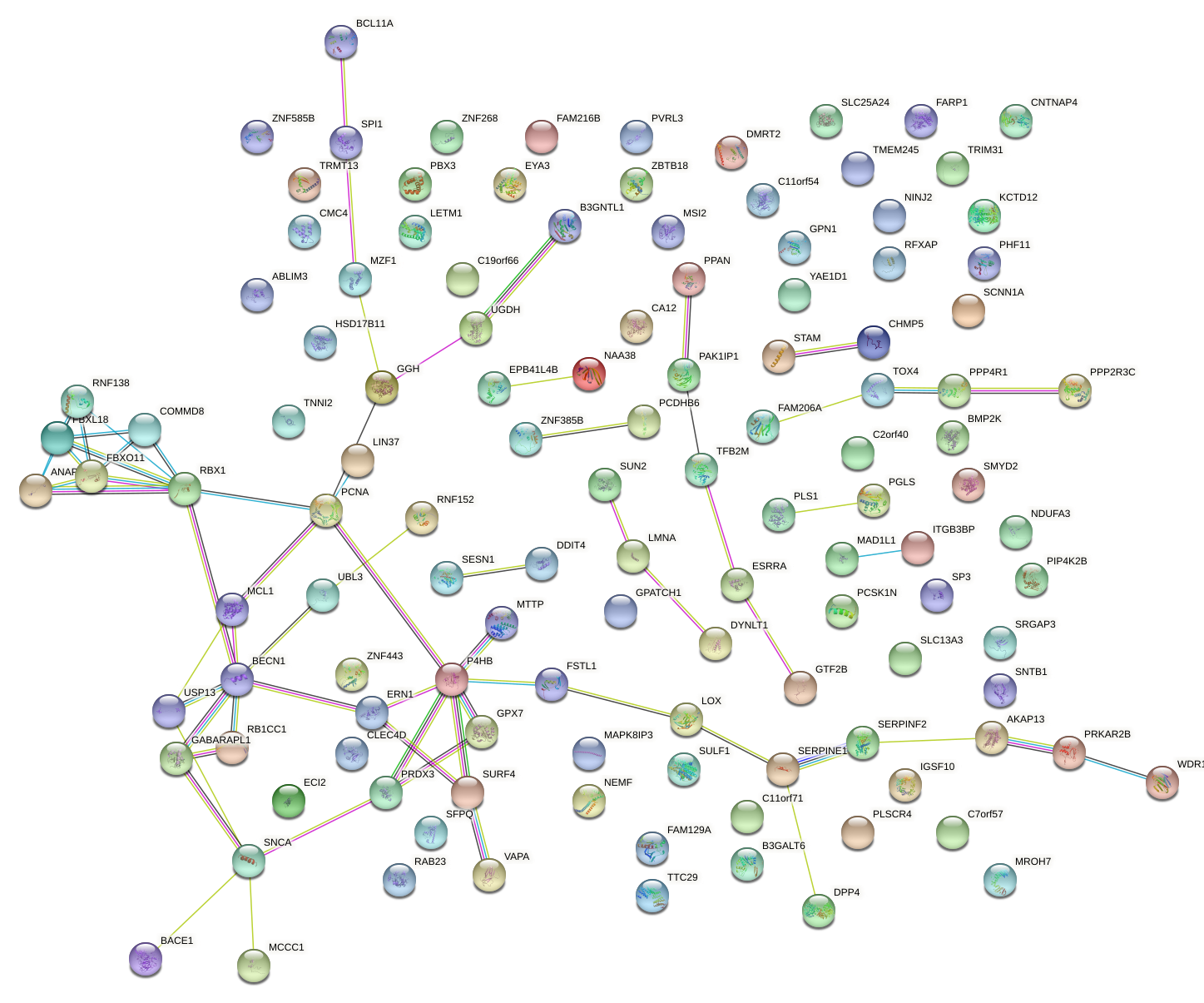


**D**


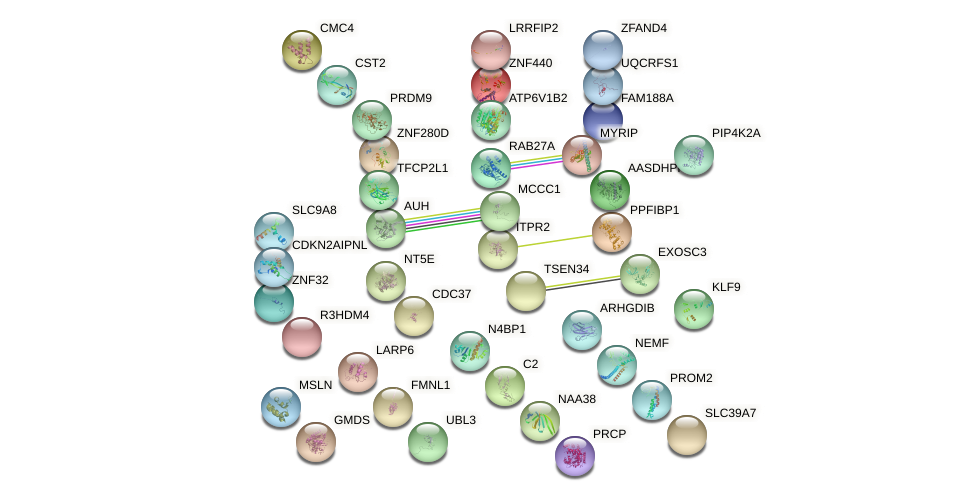


Figure S7. Modules visualized in String database. (A) Blue module visualized in String database. (B) Green module visualized in String database. (C) Turquoise module visualized in String database. (D) Yellow module visualized in String database.

**B**

**A**


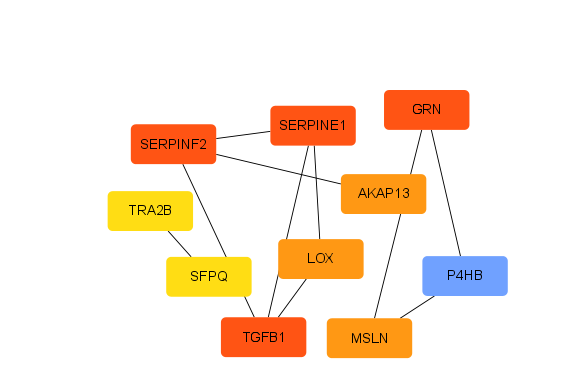

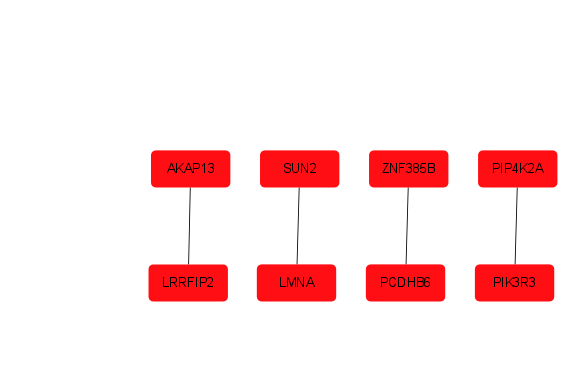


**D**

**C**


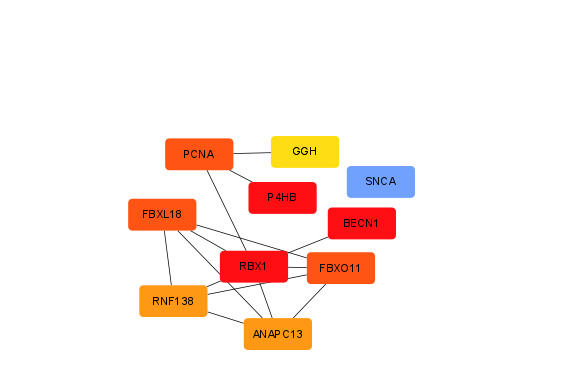

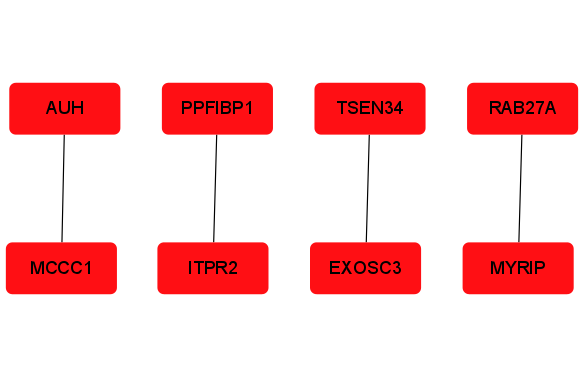


Figure S8. Top genes of modules visualized in Cytoscape. (A) Top genes in blue module visualized in Cytoscape. (B) Top genes in green module visualized in Cytoscape. (C) Top genes in turquoise module visualized in Cytoscape. (D) Top genes in yellow module visualized in Cytoscape


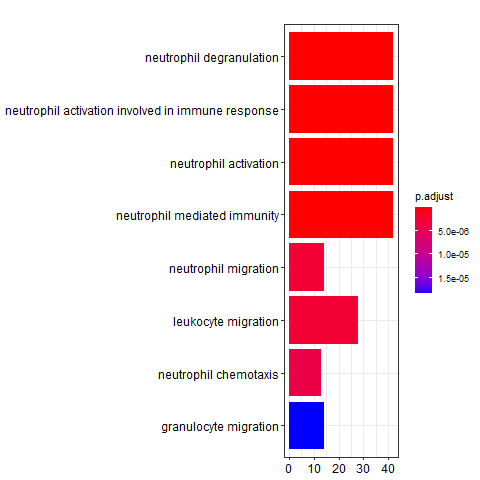

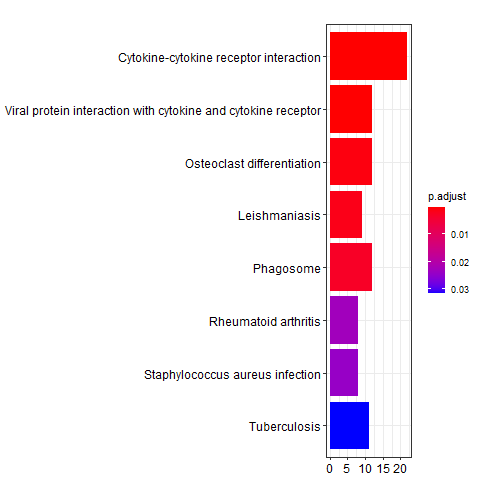


**B**

**A**

Figure S9. Results of GO and KEGG enrichment analysis of genes in modules positively related to asthma severity. (A) Results of GO enrichment analysis. (B) Results of KEGG enrichment analysis.

**A**

**C**

**B**


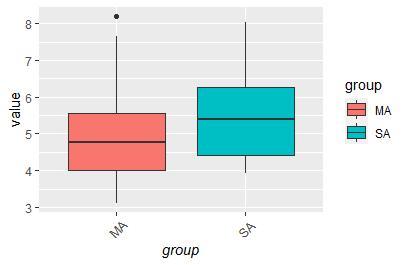

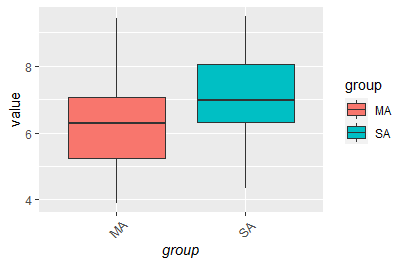

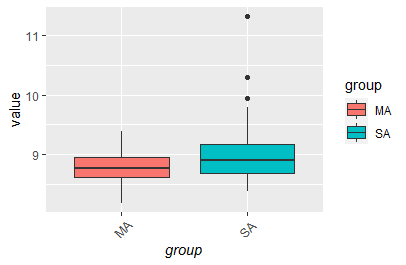


**F**

**E**

**D**


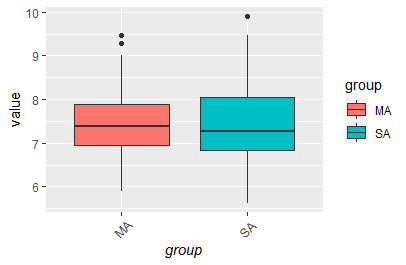

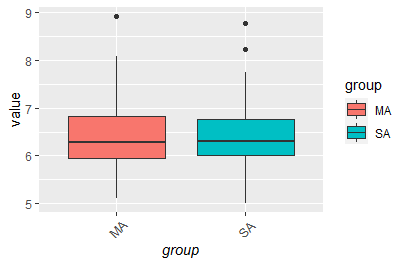

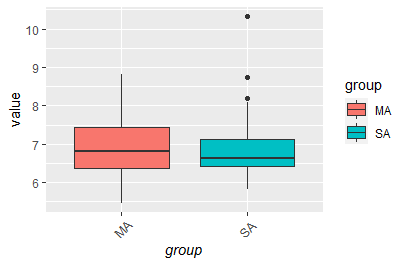


**I**

**J**

**G**


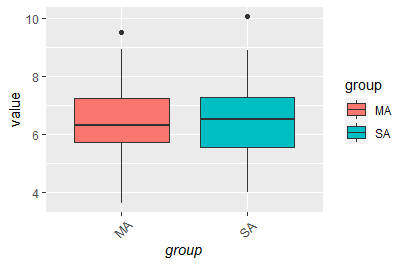

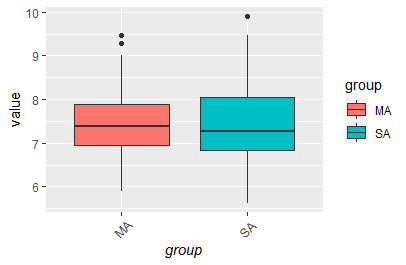

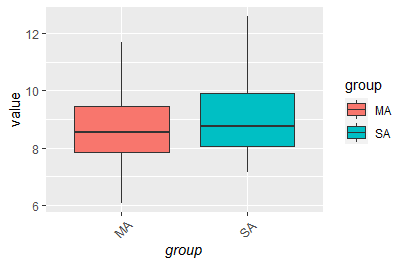


**K**


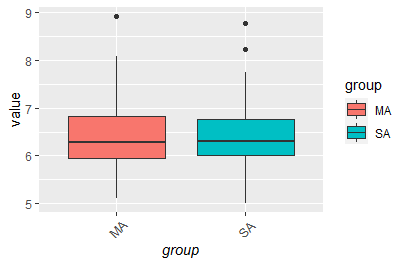


Figure S10. Expression of hub genes between mild-moderate asthma and severe asthma. (A) CXCR1 (B) CXCR2 (C) TLR2 (D) FCGR2A (E) FCGR3B (F) CCR7 (G) CCR1 (H) PLEK (I) FPR1 (J) ITGAM.

**B**

**C**

**A**


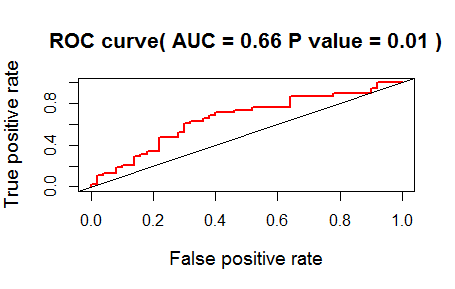

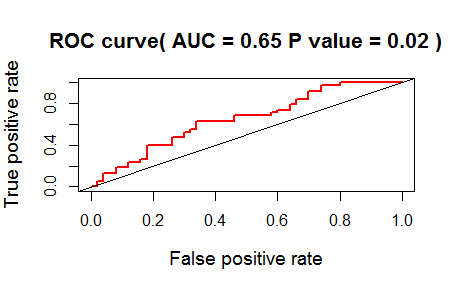

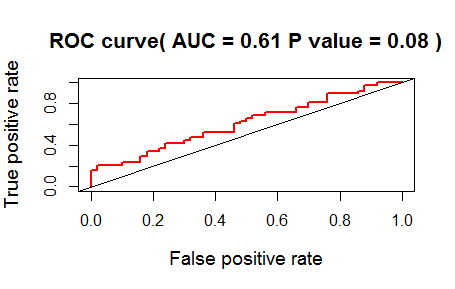


**F**

**E**

**D**


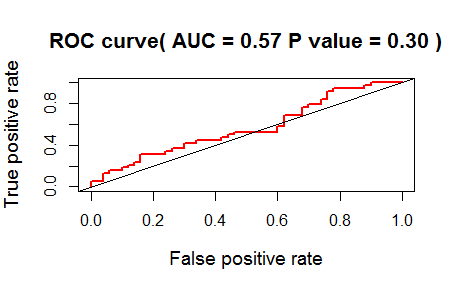

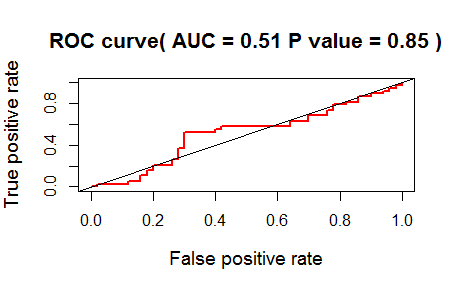

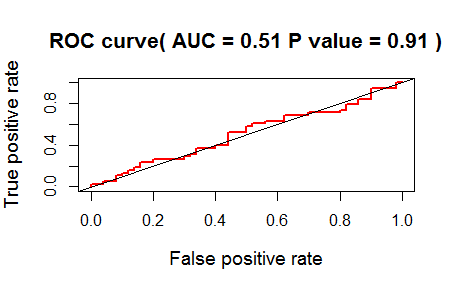


**I**

**H**

**G**


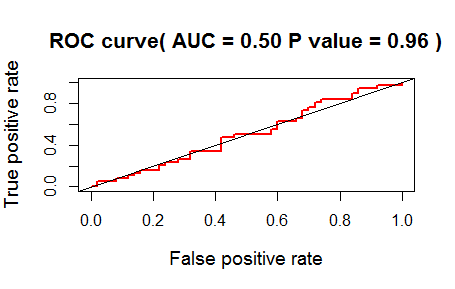

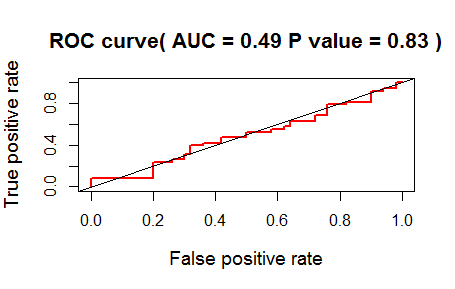

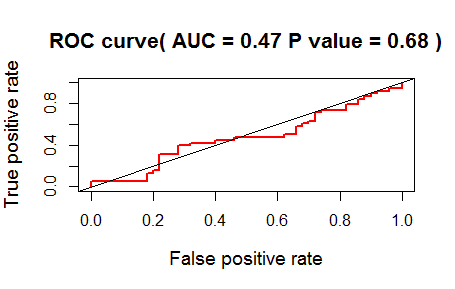

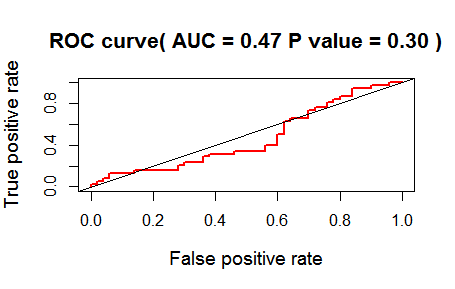


**J**

Figure S11. Receiver operating characteristics curve analyses of hub genes in GSE43696. (A) CXCR2 (B) CXCR1 (C) TLR2 (D) FPR1 (E) FCGR3B (F) CCR1 (G) ITGAM (H) PLEK (I) FCGR2A (J) CCR7

**B**

**A**


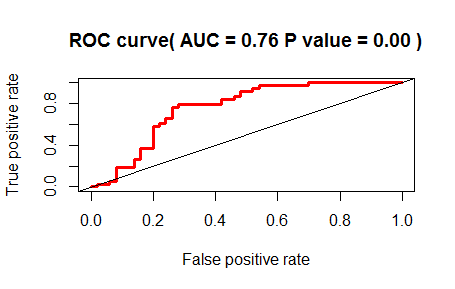

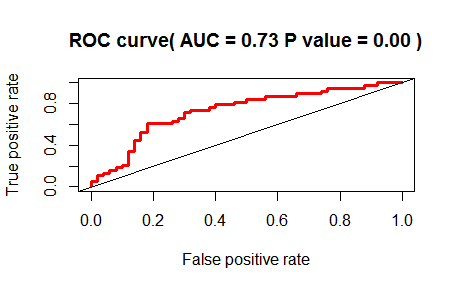


**D**

**C**


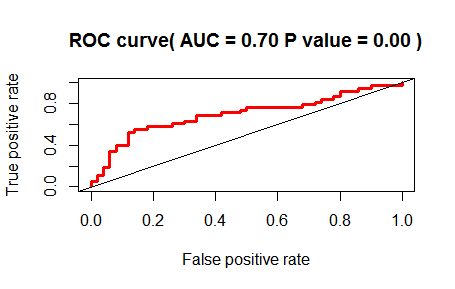

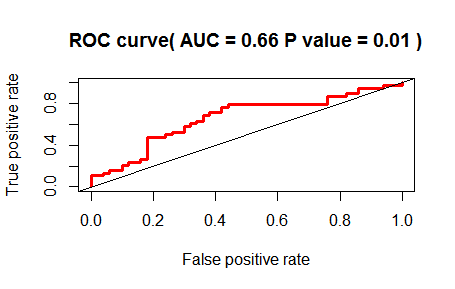


Figure S12. Receiver operating characteristics curves of combined top genes in each module. (A) Blue module (B) Green module (C) Turquoise module (D) Yellow module

Table S1. Correlations of hub genes and asthma

| Gene | Asthma susceptibility^a^ | | Asthma severity^b^ | | Asthma severity^c^ | |
| --- | --- | --- | --- | --- | --- | --- |
|  | correlation | P value | correlation | P value | correlation | P value |
| CCR1 | -0.065 | 0.634 | 0.167 | 0.217 | 0.584 | 0.003* |
| CCR7 | -0.100 | 0.463 | 0.128 | 0.348 | 0.599 | 0.002* |
| CXCR2 | 0.107 | 0.432 | 0.380 | 0.004* | 0.723 | <0.001^#^ |
| CXCR1 | 0.198 | 0.143 | 0.397 | 0.002* | 0.611 | 0.001* |
| TLR2 | -0.078 | 0.570 | 0.132 | 0.373 | 0.537 | 0.007* |
| FCGR2A | 0.086 | 0.526 | 0.307 | 0.021* | 0.696 | <0.001^#^ |
| FCGR3B | 0.067 | 0.624 | 0.365 | 0.006* | 0.681 | <0.001^#^ |
| FPR1 | 0.089 | 0.512 | 0.315 | 0.018* | 0.569 | 0.004* |
| PLEK | 0.076 | 0.578 | 0.280 | 0.036* | 0.575 | 0.003* |
| ITGAM | 0.187 | 0.167 | 0.404 | 0.002* | 0.591 | 0.002* |

Asthma susceptibility^a^ : asthma susceptibility is considered as dichotomous variable, namely control and asthma.

Asthma severity^b^ : asthma severity is considered as ranked variable, namely control, mild, moderate , and severe.

Asthma severity^c^ : asthma severity is considered as dichotomous variable, namely mild and severe. This correlation is equal to |GS| of each hub gene.

P* : P value ≤ 0.05

P^#^ : P value ≤ 0.01

Table S2. Correlation of hub genes and asthma severity in GSE43696

| Gene | Correlation | P value |
| --- | --- | --- |
| CCR1 | 0.033 | 0.762 |
| CCR7 | 0.004 | 0.973 |
| CXCR2 | 0.249 | 0.019* |
| CXCR1 | 0.251 | 0.018* |
| TLR2 | 0.264 | 0.013* |
| FCGR2A | -0.060 | 0.578 |
| FCGR3B | -0.044 | 0.684 |
| FPR1 | 0.161 | 0.134 |
| PLEK | 0.008 | 0.940 |
| ITGAM | 0.007 | 0.952 |

P* : P value ≤ 0.05

Table S3. Definitions of the Participants between GSE89809 and GSE43696

|  | GSE89809 | GSE43696 |
| --- | --- | --- |
| Healthy controls | GINA 2012 | American Thoracic Society (ATS) criteria |
| Mild asthma | Symptoms < once a day  Nocturnal symptoms < twice a month  • FEV_1_ or PEF ≥80% predicted  • Salbutamol as needed only | • FEV1 of >60% predicted  • With/without low-moderate dose inhaled CS. |
| Moderate asthma | Symptoms < once a day  Nocturnal symptoms <once a week  Asthma control score >1.5  • Salbutamol as needed only  • Low-dose inhaled steroids (<800 mcg beclometasone dipropionate equivalent)  • +/- Long acting beta-2-agonist |  |
| severe asthma | Nocturnal symptoms >once a week  Daily use of inhaled short-acting ß2-agonist  • FEV_1_ or PEF <80% of predicted or patient’s best  • High-dose inhaled steroids ( ≥800 mcg beclometasone dipropionate equivalent)  • Long acting beta-2-agonist  • +/- frequent or continuous oral corticosteroids | • Continuous use of high-dose inhaled CS and/or frequent use of oral CS  • Continuing symptoms and/or chronic airflow limitation |
